# Toxicological and biochemical basis of synergism between the entomopathogenic fungus *Lecanicillium muscarium* and the insecticide matrine against *Bemisia tabaci* (Gennadius)

**DOI:** 10.1038/srep46558

**Published:** 2017-04-20

**Authors:** Shaukat Ali, Can Zhang, Zeqing Wang, Xing-Min Wang, Jian-Hui Wu, Andrew G S Cuthbertson, Zhenfang Shao, Bao-Li Qiu

**Affiliations:** 1 Key Laboratory of Bio-Pesticide Innovation and Application, Engineering Research Center of Biological Control, South China Agricultural University, Guangzhou, 510640, P.R. China; 2 Guangdong Engineering Research Centre of Microbial Pesticides, Guangdong New Scene Biological Engineering Co. Ltd., Yangjiang, 529932, P.R. China

## Abstract

The sweetpotato whitefly *Bemisia tabaci* (Gennadius) was challenged with different combinations of matrine (insecticide) and *Lecanicillium muscarium* (entomopathogenic fungus). Our results revealed a synergistic relationship between matrine and *L. muscarium* on mortality and enzyme activities of *B. tabaci*. To illustrate the biochemical mechanisms involved in detoxification and immune responses of *B. tabaci* against both control agents, activities of different detoxifying and antioxidant enzymes were quantified. After combined application of matrine and *L. muscarium*, activities of carboxylestrease (CarE), glutathione-s-transferase (GSTs) and chitinase (CHI) decreased during the initial infection period. Acetylcholinestrase (AChE) activities increased during the entire experimental period, whereas those of superoxide dismutase (SOD), peroxidase (POD) and catalase (CAT) decreased during the later infection period. The increased mortality and suppression of enzymatic response of *B. tabaci* following matrine and *L. muscarium* application suggests a strong synergistic effect between both agents. The strong synergistic effect is possibly related to the disturbance of acetylcholine balance and changes in AchE activities of the whitefly as both matrine and *L. muscarium* target insect acetylcholine (Ach) receptors which in turn effects AchE production. Therefore, our results have revealed the complex biochemical processes involved in the synergistic action of matrine and *L. muscarium* against *B. tabaci*.

The sweetpotato whitefly *Bemisia tabaci* (Gennadius) (Hemiptera: Aleyrodidae) is a serious pest of agricultural crops in different regions of the world^1^,^2^. From the 1980s, *B. tabaci* Middle East-Asia Minor 1 (MEAM1) cryptic species (previously known as ‘B biotype’) has drastically increased in distribution. This has been attributed to the development and increase in global trade. Direct damage by *B. tabaci* occurs as a result of sucking plant sap from the phloem and secretion of honey dew which serves as a substrate for growth of sooty moulds^3^,^4^. In addition, adults can transmit more than 150 plant viruses to commercial crops^5^. Management of *B. tabaci* has been dominated by the frequent use of broad spectrum conventional chemical pesticides^6^,^7^. The consistent use of synthetic chemicals for *B. tabaci* management has resulted in environmental pollution and adverse effects on humans, mammals and other non-target organisms^8^. This injudicious use of chemicals leads to the intermission of natural biological control systems and outbreaks of *B. tabaci*^8^. All these factors have necessitated research and development of environmentally secure, biodegradable and indigenous methods for insect pest management^9^. Hence, the search for effective chemical constituents of naturally occurring entomopathogenic fungi based biopesticides or the combined use of entomopathogenic fungi along with synthetic chemicals to provide potential methods to overcome environmental pollution and resistance problems.

More than 750 fungi from over 90 species have been described as entomopathogenic. *Lecanicillium muscarium*^10^ is a well-known entomopathogenic fungus which has been commercialized for aphid and whitefly control^11^. Strains of *L. muscarium* have been isolated from aphids, scales, whiteflies and other insects in various regions of the world and have also been shown to be pathogenic against various other insects^12^. The host range of this species is quite broad and includes hemipteran insects as well as other arthropod orders. Wang *et al*.^13^ studied the virulence of six strains of *L. muscarium* against sweetpotato whitefly. Their results indicated that strain V16063, V3450 and Vp28 were virulent against *B. tabaci* having LC_50_ values of 2.57 × 10^5^, 6.03 × 10^5^ and 6.05 × 10^5^ conidia/ml respectively. Commercial preparations of *L. muscarium* have been on the market since 1980 and have been used successfully against whiteflies infesting different crops^13^.

Matrine is a quinolizidine alkaloid derived from the roots of *Sophora flavescens* and *S. alopecuroides*^14^. Matrine has been used as a traditional Chinese medicine and pesticide^15^. Matrine exhibits a variety of pharmacological and cytotoxic activities^16^. Recently, matrine has been used alone or in combination with other chemicals to control insect pests of different vegetables, fruits, flowers and tea crops in China^17^,^18^. In addition to the contact toxicity, matrine has shown antifeedant activities against Formosan subterranean termites (*Coptotermes formosanus* Shiraki) and two spotted spider mite (*Tetranychus urticae* Koch)^14^,^19^. Hwang *et al*.^20^ described the efficacy of a chemical formulation (KNI3126) based on a mixture of matrine and neem oil against different sucking insect pests and phytophagous mites confirming the toxic as well as biological action of matrine against phytophagous arthropods with different feeding habits.

Insects have a well developed defence mechanism against insecticides and natural pathogens consisting of multiple enzyme systems^21^. Cuticle degrading enzymes, multifunction oxidases (MFO), glutathione-S-transferase (GST), esterases (EST), acetylcholinestrase (AChE) and antioxidant enzymes (SOD, CAT, POD) are enzyme systems commonly involved in insect defence against toxic chemicals^22^. These enzymes play a pivotal role in detoxification and cellular antioxidant defences against the oxidative stress caused by chemicals or an invading pathogen^23^. It is well documented that insecticide poisoning and fungal infection can cause significant changes in enzymes^24^,^25^. A few studies have reported the complex changes in enzyme profiles in response to the joint application of insecticide and pathogens against insects^21^,^23^. However, detailed investigation on the effects of the synergistic action between *L. muscarium* and insecticides on insect enzyme systems has not been reported.

In this study, we aim to explain the possibility of synergistic effect between matrine and *L. muscarium* against *B. tabaci* as both of these agents affects their host through partial disruption of acetylcholine receptors. Matrine is known to target insect acetylcholine (Ach) receptors which in turn effects AchE production^16^. *Lecanicillium muscarium* can produce a secondary metabolite named bassianolide (a cyclooligomer depsipeptide) which can affect acetylcholine receptors of insect muscles reducing the production of AchE^26^,^27^. In addition, changes in activity profiles of different antioxidant and detoxifying enzymes of *B. tabaci* were also quantified as a function of the possible synergism between the entomopathogenic fungus *L. muscarium* and the insecticide matrine. Initially, the co-toxicity of matrine and *L. muscarium* infection was evaluated. Many previous synergistic studies have used only one dose level of each component. However, in the current study multiple dose levels of matrine, *L. muscarium* and their combinations were utilized for investigation. Their effects on different detoxifying as well as antioxidant enzyme activities in *B. tabaci* were also studied. The results will provide basic information on the interactions between *L. muscarium* and the insecticide. These findings will improve our knowledge concerning the mechanism by which *L. muscarium* overcomes the immune system of an insect host. The information obtained from this study will help in designing better control strategies involving insecticides and insect pathogens against *B. tabaci*.

## Results

### Virulence of matrine and *L. muscarium* alone or in combinations against *B. tabaci*

Mortality (%) of 2^nd^ instar *B. tabaci* nymphs differed significantly among different concentrations of matrine and the control after 8 days of application (*F*_5,12_ = 23.72; *P* < 0.001). The mortality data were used for probit analysis to calculate medial lethal concentrations (LC_50_) as shown in Table 1. The medial lethal concentration (LC_50_) of matrine was 0.83 ± 0.09 mg/L.

The percentage mortality of 2^nd^ instar *B. tabaci* nymphs differed significantly among different conidial concentrations of *L. muscarium* following 8 days application (*F*_5,12_ = 19.73; *P* < 0.001). The medial lethal concentration (LC_50_) of *L. muscarium* alone against *B. tabaci* nymphs was 0.16 mg/L (~5.5 × 10^5^ conidia/ml) following 8 days application (Table 1).

Combining matrine with *L. muscarium* in different ratios (CT_1_, CT_2_ and CT_3_) during bioassay-III resulted in higher mortality rates with LC_50_ values of 0.034, 0.063 and 0.21 mg/L for CT_1_, CT_2_ and CT_3_, respectively (Table 1). The co-toxicity coefficients based on initial toxicity data of matrine and *L. muscarium* were 125.99, 200 and 165.75 for CT_1_, CT_2_ and CT_3_ respectively, thus showing a synergistic interaction between matrine and *L. muscarium*.

In bioassay IV (Table 2), *B. tabaci* nymphs were first treated with different concentrations of *L. muscarium*. Following 24 h post fungal application different concentrations of matrine were then applied. The combined application resulted in a LC_50_ value of 0.02 mg/L and a co-toxicity coefficient of 223; again showing a synergistic interaction between matrine and *L. muscarium* (Table 2).

### Effect of matrine and *L. muscarium* on enzyme activities in *B. tabaci*

**Figure 1:**
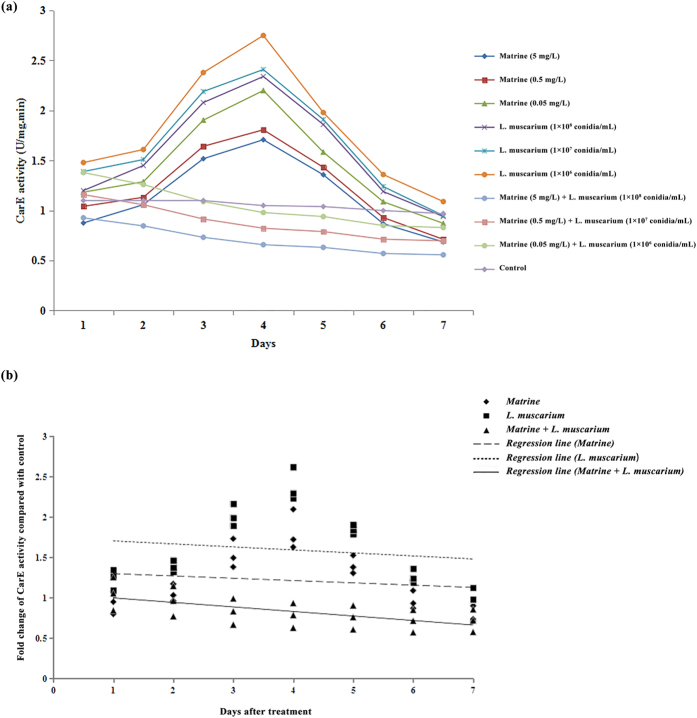
(**a**) Carboxylestrase activities of *Bemisia tabaci* at different time intervals following matrine, *Lecanicillium muscarium*, and matrine + *Lecanicillium muscarium* treatment; (**b**) Linear regression analysis between fold changes in carboxylestrase activities and different days following matrine, *Lecanicillium muscarium*, and matrine + *Lecanicillium muscarium* treatment.

The Carboxylestrease (CarE) activities of *B. tabaci* in response to different concentrations of matrine and *L. muscarium* alone or in combinations are shown in Fig. 1a. An increase in CarE activity was observed during the initial four days post matrine treatment with a sharp decline in enzyme activity being observed afterwards. *Lecanicillium muscarium* application also increased the activities of CarE in *B. tabaci* during the initial 4 days post treatment. A significant reduction in CarE activity was observed in response to the joint application of matrine and *L. muscarium* up to 7 days post treatment. The fold changes in CarE activities over control in response to different treatments and their fitted regression lines are shown in Fig. 1b. The results showed that the regression line for matrine + *L. muscarium* treatment was more sensitive than those for matrine or *L. muscarium* alone. The fitted regression lines yielded significantly different slope (*F*_2,6_ = 10.31; *P* < 0.01) and Y-intercept (*F*_2,6_ = 22.45; *P* < 0.01) values for different treatments.

**Figure 2:**
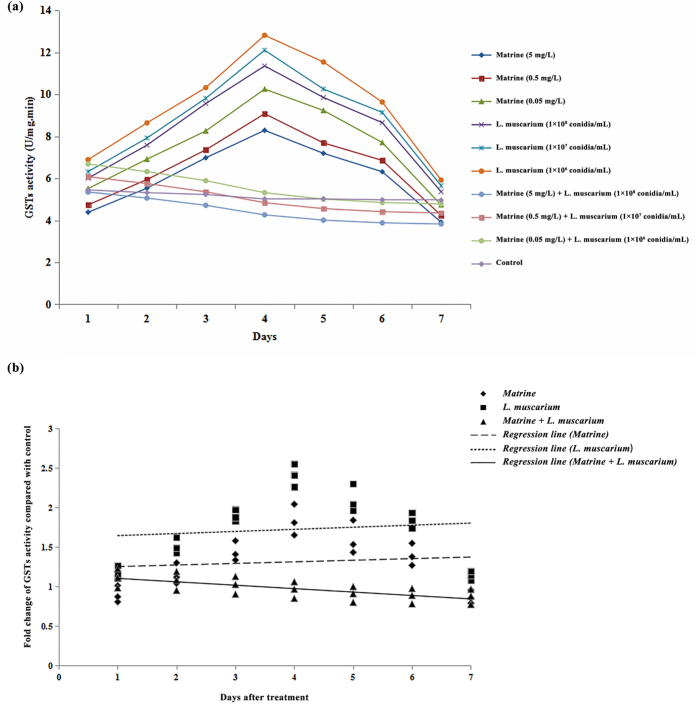
(**a**) Glutathione-s-transferase activities of *Bemisia tabaci* at different time intervals following matrine, *Lecanicillium muscarium*, and matrine + *Lecanicillium muscarium* treatment; (**b**) Linear regression analysis between fold changes in glutathione-s-transferase activities and different days following matrine, *Lecanicillium muscarium*, and matrine + *Lecanicillium muscarium* treatment.

The activities of GSTs in *B. tabaci* when treated with matrine increased up to 4 days post treatment followed by a decrease in enzyme activity during the later periods. GSTs activity also increased during the initial 4 days following *L. muscarium* treatment. The GSTs activity in *B. tabaci* decreased significantly throughout the experimental period in response to the joint application of matrine and *L. muscarium* (Fig. 2a). The fold changes in GSTs activities over the control in response to different treatments and their fitted regression lines are shown in Fig. 2b. The results showed that the regression line for matrine + *L. muscarium* treatment was more sensitive than those for matrine or *L. muscarium* alone. The fitted regression lines yielded significantly different slope (*F*_2,6_ = 16.42; *P* < 0.01) and Y-intercept (*F*_2,6_ = 19.91; *P* < 0.01) values for different treatments.

**Figure 3:**
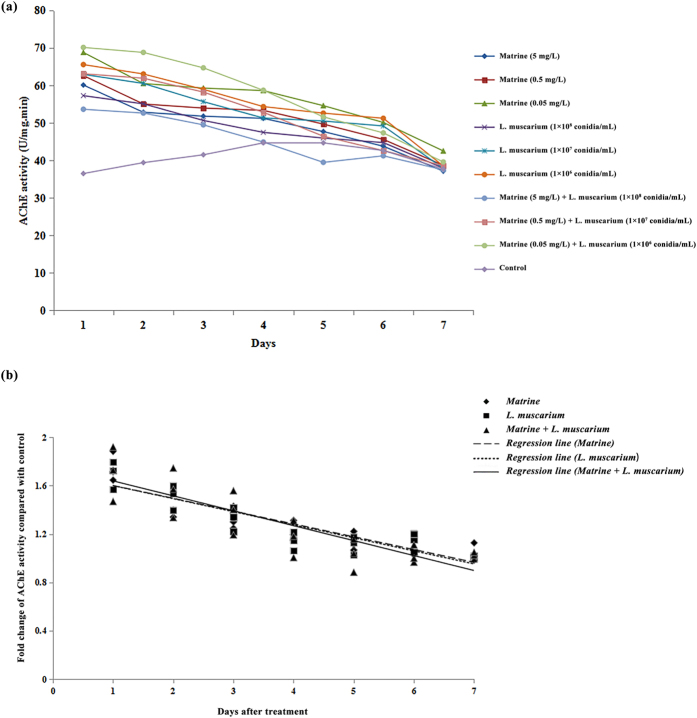
(**a**) Acetylcholinestrase activities of *Bemisia tabaci* at different time intervals following matrine, *Lecanicillium muscarium*, and matrine + *Lecanicillium muscarium* treatment; (**b**) Linear regression analysis between fold changes in acetylcholinestrase activities and different days following matrine, *Lecanicillium muscarium*, and matrine + *Lecanicillium muscarium* treatment.

Acetylcholinestrase (AChE) activity decreased in *B. tabaci* during the entire experimental period following treatment with matrine alone. When treated with *L. muscarium* alone, AChE activity in *B. tabaci* decreased throughout the experimental period. AChE activity also decreased during the whole experimental period after *B. tabaci* was treated with a mixture of matrine and *L. muscarium* (Fig. 3a). The fold changes in AchE activities over the control in response to different treatments and their fitted regression lines are shown in Fig. 3b. The results showed that the regression line for different treatments were similar to each other. The fitted regression lines yielded significantly similar slope (*F*_2,6_ = 15.36; *P* = 0. 54) and Y-intercept (*F*_2,6_ = 14.39; *P* = 0.61) values for different treatments.

**Figure 4:**
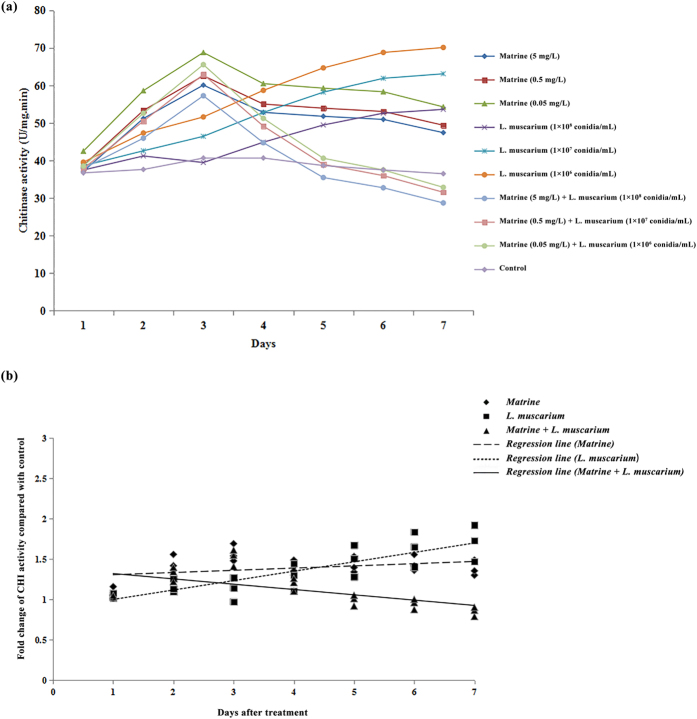
(**a**) Chitinase activities of *Bemisia tabaci* at different time intervals following matrine, *Lecanicillium muscarium*, and matrine + *Lecanicillium muscarium* treatment; (**b**) Linear regression analysis between fold changes in chitinase activities and different days following matrine, *Lecanicillium muscarium*, and matrine + *Lecanicillium muscarium* treatment.

The activities of chitinase (CHI) in *B. tabaci* increased during the initial 3 days of the experiment when whiteflies were treated with matrine alone. When whiteflies were treated with *L. muscarium* alone, the enzyme activities increased throughout the experimental period. When whiteflies were treated with a combination of matrine and *L. muscarium*, enzyme activities increased during the initial 3 days and then decreased during the later time period (Fig. 4a). The fold changes in CHI activities over the control in response to different treatments and their fitted regression lines are shown in Fig. 4b. The results showed that the regression line for matrine + *L. muscarium* treatment was more sensitive than those for matrine or *L. muscarium* alone. The fitted regression lines yielded significantly different slope (*F*_2,6_ = 25.49; *P* < 0.001) and Y-intercept (*F*_2,6_ = 17.56; *P* < 0.01) values for different treatments.

**Figure 5:**
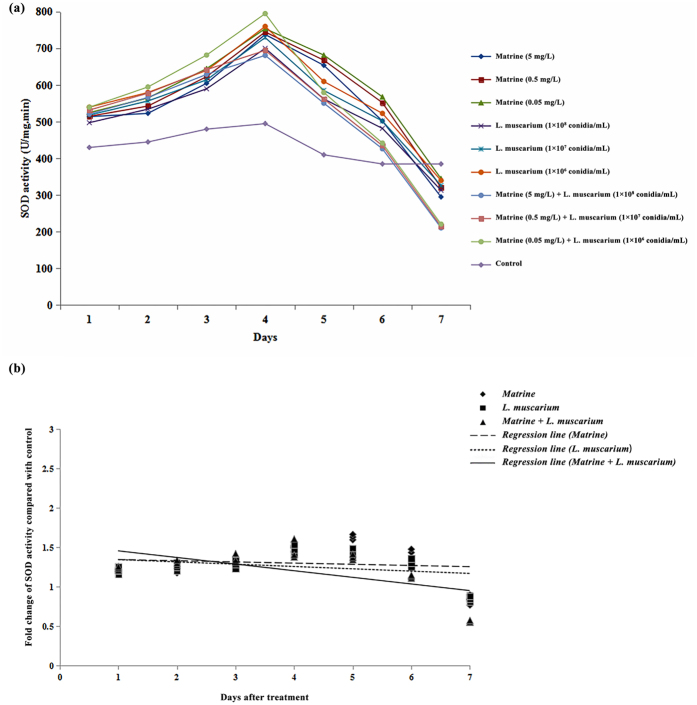
(**a**) Superoxide dismutase activities of *Bemisia tabaci* at different time intervals following matrine, *Lecanicillium muscarium*, and matrine + *Lecanicillium muscarium* treatment; (**b**) Linear regression analysis between fold changes in superoxide dismutase activities and different days following matrine, *Lecanicillium muscarium*, and matrine + *Lecanicillium muscarium* treatment.

**Figure 6:**
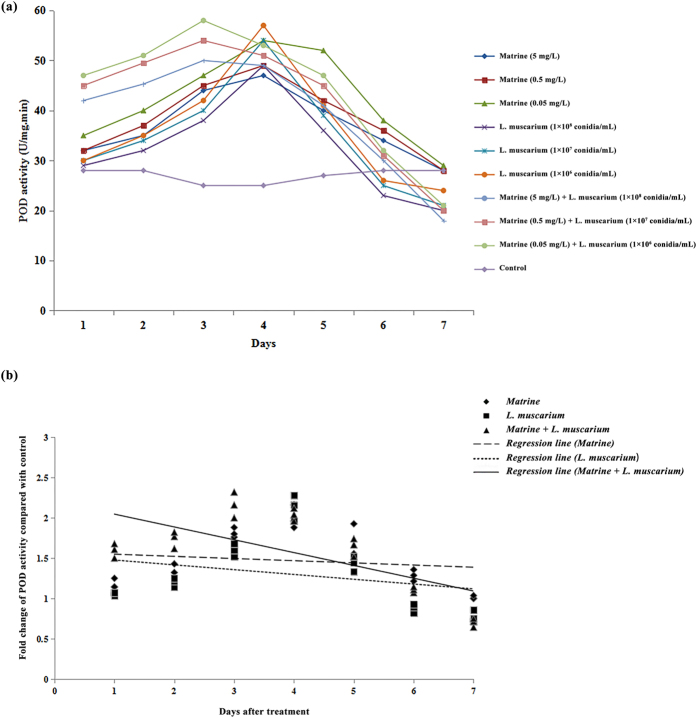
(**a**) Peroxidase activities of *Bemisia tabaci* at different time intervals following matrine, *Lecanicillium muscarium*, and matrine + *Lecanicillium muscarium* treatment; (**b**) Linear regression analysis between fold changes in peroxidase activities and different days following matrine, *Lecanicillium muscarium*, and matrine + *Lecanicillium muscarium* treatment.

**Figure 7:**
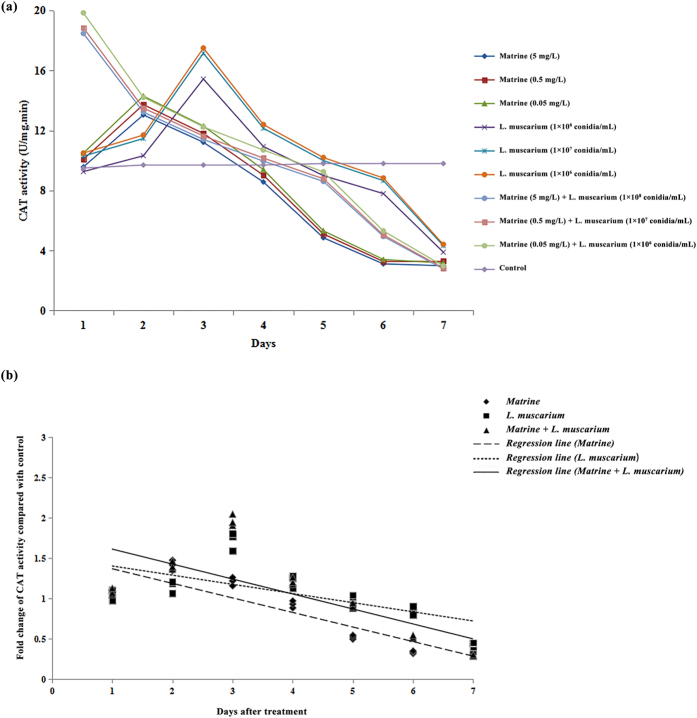
(**a**) Catalase activities of *Bemisia tabaci* at different time intervals following matrine, *Lecanicillium muscarium*, and matrine + *Lecanicillium muscarium* treatment; (**b**) Linear regression analysis between fold changes in catalase activities and different days following matrine, *Lecanicillium muscarium*, and matrine + *Lecanicillium muscarium* treatments.

The activities of different antioxidant enzymes (SOD, POD and CAT) in *B. tabaci* increased during the initial 4 days after treatment with matrine or *L. muscarium* alone. When whiteflies were treated with a mixture of matrine and *L. muscarium*, activities of the antioxidant enzymes (SOD, POD and CAT) increased for up to 3 days. At the end of the experimental period the enzyme activities observed were significantly lower than the control (Figs 5a–7a). The fold changes in activities of the different antioxidant enzymes (SOD, POD and CAT) over the control in response to different treatments and their fitted regression lines are shown in Figs 5b–7b. The results showed that the regression lines for matrine + *L. muscarium* treatment were more sensitive than those for matrine or *L. muscarium* alone for SOD and POD while in case of CAT the fitted regression line of matrine was more sensitive when compared with the other treatments. The fitted regression lines yielded significantly different slope (SOD: F_2,6_ = 23.65, *P* < 0.001; POD: *F*_2,6_ = 14.27, *P* < 0.01 and CAT: *F*_2,6_ = 15.19, *P* < 0.01) and Y-intercept (SOD: *F*_2,6_ = 19.63, *P* < 0.01; POD: *F*_2,6_ = 13.22, *P* < 0.01 and CAT: *F*_2,6_ = 25.78, *P* < 0.01) values for different treatments.

## Discussion

Development of integrated control programs against *B. tabaci* involving *L. muscarium* and chemical pesticides requires a clear understanding concerning the effects of chemicals on the physiology and pathogenicity of entomopathogenic fungi^28^. There have been a few *in vivo* studies which have indicated that combinations of *L. muscarium* and insecticides can have synergistic, antagonistic or additive mortality effects against *B. tabaci*^29^,^30^. However, most of the studies reporting on *L. muscarium*-insecticide interactions have shown the insect mortality or LC_50_ values by combining a single dose of fungi or chemical. We believe this is not an appropriate approach to determine the most effective synergistic formulation^23^,^31^. In this study Sun and Johnson’s co-toxicity coefficient was used to estimate the efficacy of different matrine and *L. muscarium* combinations (prepared at different ratios) against *B. tabaci*. We observed synergistic interactions when 2^nd^ instar nymphs of *B. tabaci* were treated with different doses of matrine and *L. muscarium*. All the combined treatments of matrine and *L. muscarium* (CT_1_, CT_2_, CT_3_ in bioassay III and CT_4_ in bioassay IV) had higher rates of *B. tabaci* mortality (>93%) when compared with the mortality of *B. tabaci* in response to matrine or *L. muscarium* applied alone. The combined treatments CT_1_, CT_2_, CT_3_ (in bioassay III) and CT_4_ (in bioassay IV) had synergistic interactions with co-toxicity coefficients of 125.99, 200, 165.75 and 223 respectively. These findings illustrate the possible effectiveness of dual attack strategy for whitefly management. Our results showed that matrine can improve the efficacy of *L. muscarium* against *B. tabaci* during the infection period. The observed synergistic effect can be related to the target site of action of matrine and the toxin produced by *L. muscarium*. Both agents attack acetylcholine production by the insect which makes their interaction strongly synergistic, although the strength of this synergistic interaction can vary with the proportion of both agents applied, as well as, the timing of their application. This study has also shown possible physiological or biochemical consequences of the joint matrine and *L. muscarium* application against *B. tabaci*. The enzymatic response of *B. tabaci* observed was of a complex nature as the immune system of the whitefly was simultaneously trying to detoxify the insecticide as well as defend against fungal attack. The enzyme quantified during this study showed moderate to high variation in response to matrine and *L. muscarium* application.

In insects, detoxification of insecticides or pathogens is mainly accomplished by carboxylesterases (CarE) and glutathione-S-transferase (GSTs)^32^. Our findings showed an increase in CarE and GSTs activities during the initial 4 days following matrine treatment. This increase in enzyme activity in response shows the possible involvement of these enzymes in the insecticide detoxification mechanism of *B. tabaci*^23^. Luo & Zhang^33^ also observed similar fluctuation of CarE and GSTs in *Plutella xylostella* in response to matrine treatment. Matrine treatment also resulted in inhibition of CarE in turnip aphid (*Lipaphis erysimi*)^34^. Although few reports are available on changes in CarE and GSTs activity following matrine treatment, the extent of changes can vary with change in the insect species targeted and the concentration of matrine used. In this study, we found that CarE and GSTs increased during initial periods following *L. muscarium* treatment which is similar to the findings of Tian *et al*.^35^ who also observed similar changes in CarE and GSTs activity of *B. tabaci* following *Isaria fumosorosea* applications. They observed increases in activity of the said enzymes until 72 h post fungal application after which enzyme activities were restrained leading to metabolic imbalance and insect death^35^. The treatment of *B. tabaci* with a combination of matrine and *L. muscarium* resulted in a significant reduction of CarE and GSTs activities throughout the experimental period. These results are in line with the findings of Jia *et al*.^23^ who have shown a similar reduction of enzyme activity in *Locusta migratoria* following joint application of chlorantriniliprole and *Metarhizium anisopliae*. The changes in activities of detoxifying enzymes explained above can make the target pest more susceptible to fungal infection^36^. The reduction in CarE and GSTs activities in response to joint application of insecticide (matrine) and the entomopathogenic fungi (*L. muscarium*) throughout the experimental period can be related to the sequence of chemical’s/pathogen’s action against *B. tabaci*. Insecticides normally act as a stressor increasing the susceptibility of a target pest to an entomopathogenic fungus^37^. The enzyme acetylcholinestrase (AchE) rapidly terminates nerve impulses by catalyzing the hydrolysis of the neurotransmitter, acetylcholine at peripheral and central synapses of the insects’ nervous system^38^. AchE is also an important target site for insecticide action in the central nervous system of insects^39^. During this study, AchE activities of *B. tabaci* decreased throughout the experimental period following treatment with matrine and *L. muscarium* alone or in combination. The decrease in AchE activities can be related to the mode of action of both agents. Matrine, an alkaloid extracted from *S. flavescens* is known to target insect acetylcholine (Ach) receptors which in turn effects AchE production^40^. Luo *et al*.^34^ observed similar inhibition of AchE activity in turnip aphid (*L. erysimi*) following matrine application. The reduction in AchE activity of *B. tabaci* following *L. muscarium* application is in line with the findings of Zibaee *et al*.^24^ who observed similar inhibition of AchE activity when *Beauveria bassiana* and its secondary metabolites were applied against sunn pest (*Eurygaster integriceps*). These changes in AchE activity of *B. tabaci* can be related to the production of a secondary metabolite named bassianolide by *L. muscarium*. Bassianolide can inhibit acetylcholine receptors of insect muscles reducing the production of AchE^26^,^27^.

Chitinase (CHI) is an important component of insect growth and development. Chitinases degrade chitin present in an insect cuticle and peritrophic membrane^41^. Chitinases have also been reported to be involved in insect defence against entomopathogenic fungi and parasites^42^. Our results showed an increase in CHI activitythroughout the experimental period following *L. muscarium* application whereas reduction in CHI activities was observed after 3 days of matrine application or joint application of matrine and *L. muscarium*. We speculate that the joint application of matrine and *L. muscarium* disrupted the insect moulting process by creating an imbalance of chitinase production which is an essential enzyme required for moulting^23^.

Antioxidant enzymes (SOD, CAT, and POD) are also called enzymes of the protective cellular system as superoxide dismutase can enhance H_2_O_2_ production from O_2_ through dismutation while catalase and peroxidase are known to catalyze H_2_O production from H_2_O_2_. These reactions can result in the elimination of bio-membrane damage by reactive oxygen species (ROS)^43^. In our study, the activities of antioxidant enzymes (SOD, CAT, and POD) increased during the initial 3 days followed by a decrease when treated with *L. muscarium* alone. On the other hand, when matrine was applied in combination with *L. muscarium*, SOD and POD activities increased during the initial 3 days but CAT activities started to decrease after 24 h. This decrease in CAT activity can have a direct relationship with the accumulation of superoxide radicals produced during the destruction process^23^. The decreased activities of antioxidant enzymes (SOD, CAT, and POD) during the later experimental period can result in a reduced elimination of ROS which in turn can denature different biomolecules of the insect body. The denaturation of biomolecules can stop all the cellular processes, so leading to the death of the insect^44^.

To summarize, the enzymatic response of *B. tabaci* to combined matrine and *L. muscarium* treatment displays a reduction in CarE, GSTs and CHI during the initial infection period, whereas SOD, POD and CAT activities decreased during the later infection period. The changes in enzymatic activities suggest that the probability of *L. muscarium* infection was increased by matrine and *L. muscarium* which in turn enervated the whitefly defence against matrine. Based on the results of current and previous studies, we can hypothesize that the enzymatic defence of *B. tabaci* is activated by the assault of either matrine or *L. muscarium* and that the combination of these two agents can overcome this defence strategy through a strong synergistic effect. The chemical basis of this strong synergistic effect is possibly related to the disturbance of the acetylcholine balance and changes in AchE activities of the whitefly as both matrine and *L. muscarium* can target insect acetylcholine (Ach) receptors which in turn effects AchE production^24^,^40^. Therefore, our results have revealed the complex biochemical processes involved in the synergistic action of matrine and *L. muscarium* against *B. tabaci*. These findings will be helpful in designing effective means of integrated whitefly control in the future.

## Materials and Methods

### Insect cultures

MEAM1 whitefly were collected in Guangzhou from cotton plants and reared at the Engineering Research Center of Biological Control, Ministry of Education, South China Agricultural University. The MEAM1 *B. tabaci* was identified by using mitochondrial COI (mtCOI) sequencing as described by Khasdan *et al*.^45^. MEAM1 *B. tabaci* was reared in a glasshouse on *Gossypium hirsutum* (Malvaceae), Lu-Mian 32 under the following conditions 26 ± 1 °C, 70 ± 10% R.H. and 14:10-h light: dark photoperiod.

### Fungus and insecticides

For all assays, the fungal strain *L. muscarium* V20 originally isolated from *Trialeurodes sp*., deposited to the collection at Key laboratory of biopesticides innovation and application of Guangdong Province, South China Agricultural University, Guangzhou, P.R. China was used during these studies.

Matrine powder (95%) was provided by Guangdong New Scene Bioengineering, Yangjiang, China. Preliminary trials conducted to test the possible influence of matrine on fungal growth and fungal enzymes showed no positive or negative effect on fungal growth and enzyme production (Ali *et al*. unpublished data).

### Bioassays

#### Bioassay I: Efficacy of *L. muscarium* against *B. tabaci*

Five different conidial concentrations of *L. muscarium* (1 × 10^4^, 1 × 10^5^, 1 × 10^6^, 1 × 10^7^ and 1 × 10^8^ conidia/ml) were prepared by following the method of Ali *et al*.^46^. The fungal conidia cultured on Potato Dextrose Agar (PDA) were harvested with deionized water containing 0.01% Tween 80 and sieved using filter paper (Whatman No. 2; Science Kit & Boreal Laboratories, New York, NY, USA) into sterile vials. Conidia were counted using a compound microscope and a hemocytometer (0.0625 m^2^; Fuchs-Rosenthal Merck Euro Lab, Darmstadt, Germany) to calibrate a suspension of 1 × 10^8^ conidia/ml. Spore viability was determined before preparation of suspension by spreading 0.2 ml of suspension on PDA and estimating the number of germinated propagules after 24 h of incubation at room temperature. Spores were considered viable when the germ tube length was equal to or greater than the width. The viability of conidia was assessed immediately before each experiment was started and germination was estimated to >95% for all experiments. The conidial suspension (1 × 10^8^ conidia/ml) was used as a stock solution and lower concentrations (1 × 10^7^, 1 × 10^6^, 1 × 10^5^ and 1 × 10^4^ conidia/ml) were prepared through serial dilutions by using deionized water containing 0.01% Tween 80.

Newly molted 2^nd^ instar *B. tabaci* nymphs were treated with different concentrations by dipping the infested leaves^47^ in each conidial concentration for 30 s, and then removing them to air dry before being transferred to clean glass petri dishes (ø 9 cm). Control insect groups were treated with deionized water containing 0.01% Tween 80. A piece of filter paper was placed at the bottom of the dish with 200 μl water for moisture maintenance. Each treatment (each conidial concentration) and control was repeated three times with fresh batch of insects and fresh conidial suspension. Each repetition contained four leaves with 100 individuals per leaf. Mortality was recorded at 24 h intervals for 8 days. Infected nymphs were identified by their red colour and later on by outgrowth of mycelia while naturally dead nymphs were yellowish in colour with flattened bodies. The cadavers were removed and cultured separately at 26 °C and >90% R.H. to observe the fungal sporulation. If sporulation was observed, death was considered as a result of *L. muscarium* infection.

To obtain *L. muscarium* infected whitefly nymphs for enzymatic studies, *B. tabaci* nymphs were treated with three conidial concentrations (1 × 10^6^, 1 × 10^7^ and 1 × 10^8^ conidia/ml) by following the method used above.

#### Bioassay II: Efficacy of matrine against *B. tabaci*

Matrine (95% pure) was dissolved in ddH_2_O to prepare a stock solution of 50 mg/L. Five different concentrations of matrine (15 mg/L, 5 mg/L, 0.5 mg/L, 0.05 mg/L and 0.005 mg/L) were prepared by serial dilutions. Newly molted 2^nd^ instar *B. tabaci* nymphs were treated with different concentrations by dipping the infested leaves in each concentration for 30 s, and then removing them to air dry before being transferred to clean glass petri dishes (ø 9 cm). Control insect groups were treated with ddH_2_O. A piece of filter paper was place at the bottom of the dish with 200 μl water for moisture maintenance. Each treatment (each concentration) and control was repeated three times with a fresh batch of insects. Each repetition contained four leaves with 100 individuals per leaf. Mortality was recorded at 24 h intervals for 8 days. Infected nymphs were identified by their red colour while naturally dead nymphs were yellowish in colour with flattened bodies. To obtain insecticide challenged whitefly nymphs for enzymatic studies, *B. tabaci* nymphs were treated with three concentrations of matrine (5, 0.5 and 0.05 mg/L) by following the method used above.

#### Bioassay III: Efficacy of *L. muscarium* combined with matrine against *B. tabaci*

*Lecanicillium muscarium* was combined with matrine in four dose combinations for each of three different proportions (Table 3). Newly molted 2^nd^ instar *B. tabaci* nymphs were treated with different concentrations by again dipping the infested leaves in each concentration for 30 s, and then removing them to air dry before being transferred to clean glass petri dishes (ø 9 cm). Control insect groups were treated with deionized water containing 0.01% Tween 80. A piece of filter paper was placed at the bottom of the dish with 200 μl water to maintain moisture. Each treatment (each dose combination) and control was repeated three times with both a fresh batch of insects and conidial suspension. Each repetition contained four leaves with 100 individuals per leaf. Mortality was recorded at 24 h intervals for 8 days. Infected nymphs were identified by their red colour and later on by outgrowth of mycelia while naturally dead nymphs were yellowish in colour with flattened bodies. To obtain *L. muscarium* + matrine challenged whitefly nymphs for enzymatic studies, *B. tabaci* nymphs were treated with three dose combinations (5 mg/L + 1 × 10^8^ conidia/ml; 0.5 mg/L + 1 × 10^7^ conidia/ml; and 0.05 mg/L + 1 × 10^6^ conidia/ml) by following the method used above.

#### Bioassay IV: Susceptibility of *B. tabaci* to matrine after 24 h exposure to *L. muscarium*

Newly molted 2^nd^ instar *B. tabaci* nymphs were treated with different *L. muscarium* concentrations as shown in Table 3 and as described above. Treated nymphs on leaves were placed into clean glass petri dishes (ø 20 cm). A piece of filter paper was placed at the bottom of the dish with 200 μl of water to maintain moisture. After 24 h, leaves bearing whitefly nymphs infected with *L. muscarium* were dipped in different concentrations of matrine (shown in Table 3) for 30 s. Each treatment (each dose combination) and control was repeated three times with both a fresh batch of insects and conidial suspension. Each repetition contained four leaves with 100 individuals per leaf. Mortality was recorded at 24 h intervals for 8 days. Infected nymphs were identified by their red colour and later on by outgrowth of mycelia while naturally dead nymphs were yellowish in colour with flattened bodies.

### Enzyme preparation

Second instar whitefly nymphs (100 individuals) inoculated with different doses of *L. muscarium*, matrine, and *L. muscarium* + matrine dose-combinations were removed from leaf surfaces on a daily basis for a period of 7 days. Collected nymphs were homogenized with ice cold 0.05 M Sodium phosphate buffer at different pH levels as follows: pH 7.0 (AchE and CarE: 1.5 ml PBS), pH 7.3 (SOD, POD and CAT: 1.5 ml PBS) and pH 7.5 (CHI and GSTs: 1.5 ml PBS). The homogenized samples were centrifuged at 12,000 rpm for 10 min at 4 °C. The supernatants were transferred to new tubes and centrifuged at 12,000 rpm for 15 min at 4 °C. The final supernatants were used as the enzyme preparation for the different enzyme and protein assays.

### Protein assay

Total protein contents of supernatants from insect homogenates were quantified following Bradford^48^ by using bovine albumin serum (BSA) as standard.

### Enzyme activity assays

Carboxylestrease (CarE) was quantified by using 1- naphthyl acetate as substrate^49^. The reaction mixture (0.45 ml 0.05 mol/L sodium phosphate buffer, 1.8 ml 0.00003 mol/L 1- naphthyl acetate and 0.05 ml sample) was incubated in water at 30 °C for 15 min followed by addition of 0.9 ml 1% fast blue RR salt to terminate the reaction. The change in absorbance was measured at 600 nm. Enzyme activity was determined by the protein changes per unit over time.

The glutathione S- transferase (GSTs) was conducted using the procedures developed by Habig^50^. The changes in absorbance were measured at 340 nm. The enzyme activity was measured by protein changes per unit over time (U/mg per min).

Acetylcholinestrase (AChE) activity was assayed by following Ellman *et al*.^51^ with some slight modifications. The reaction mixture contained 50 μL sample solution, 100 μL 45 μM 5-5-dithiobis-(2-nitrobenzoic acid), 100 μL acetylthiocholine iodide and 90 μL sodium phosphate buffer. The change in absorbance at 405 nm was recorded for 40 min. Enzyme activity was calculated as the rate of absorbancechange per mg protein (mOD/min/mg).

Superoxide dismutase (SOD) activity was measured in supernatants by nitro blue tetrazolium (NBT) reduction^52^. One unit of SOD activity was defined as the amount of SOD required for inhibition of the reduction of NBT by 50% (A_560_) and was expressed as units per mg protein (U/mg protein).

Catalase activity was assayed by the method described by Beers & Sizer^53^, in which the decomposition of hydrogen peroxide (H_2_O_2_) was analyzed spectrophotometrically at 240 nm. One unit of catalase activity was defined as the amount of enzyme that decomposes1 mmol H_2_O_2_/min at an initial H_2_O_2_ concentration of 30 mM at pH 7.0 and 25 °C.

The activity of peroxidase (POD) was assessed by following the method of Simon *et al*.^54^. The changes in absorbance were measured at 470 nm. Enzyme activity was determined by the protein changes per unit (U/min per min).

The chitinase (CHI) activity in the culture supernatant was estimated as described earlier using acid swollen chitin as the substrate. To prepare acid swollen chitin, the chitin (10 g), was suspended in chilled *O*-phosphoric acid (88%, w/v) and left at 0 °C for 1 h with stirring. The acid swollen chitin was repeatedly washed with chilled distilled water, followed with a 1% (w/v) NaHCO_3_ solution which was further dialyzed against cold distilled water. After homogenization in a Waring^®^ blender (1 min), 50 mM acetate buffer, pH 5.0, was added to the suspension so that 1 ml of suspension contained 7 mg of chitin. The reaction mixture for the chitinase assay contained 1 ml 0.7% acid swollen chitin, 1 ml 50 mM acetate buffer, pH 5.0 and 1 ml enzyme solution that was incubated at 50 °C for 1 h. The *N*-acetylglucosamine (GlcNAc) produced was estimated colorimetrically with *p*-dimethyl amino benzaldehyde (DMAB)^55^. One international unit was defined as the activity that produced 1 μmol of GlcNAc per min.

### Data analysis

Median lethal concentration (LC_50_) values for each bioassay were calculated through probit analysis in SAS 9.1 software for windows^56^. LC_50_ values for different bioassays were subjected to one –way ANOVA at 5% level of significance followed by mean comparison through Tukey’s HSD test.

Coefficients of co-toxicity were calculated by following Sun & Johnson^57^. The co-toxicity coefficients for the mixed formulation were calculated after calculating the LC_50_ of each component in the mixture. Calculations were performed by using the following equations (Equations 1) described by Jia *et al*.^23^:


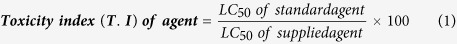



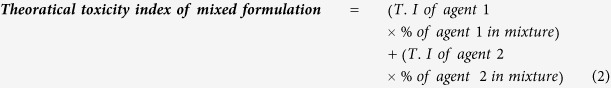



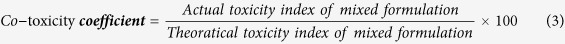


Co-toxicity coefficient greater than 100 showed a synergistic effect while the mixture with co-toxicity coefficient less than 100 corresponded to antagonistic effect. A Co-toxicity coefficient of 100 indicated that the effect of the mixture was similar to that predicted from the proportions of the two components.

In the figures presented, each point represents the average x-fold change in values from five sub-replicates of enzyme activities compared with the control.

## Additional Information

**How to cite this article**: Ali, S. *et al*. Toxicological and biochemical basis of synergism between the entomopathogenic fungus *Lecanicillium muscarium* and the insecticide matrine against *Bemisia tabaci* (Gennadius). *Sci. Rep.* **7**, 46558; doi: 10.1038/srep46558 (2017).

**Publisher's note:** Springer Nature remains neutral with regard to jurisdictional claims in published maps and institutional affiliations.

## Figures and Tables

**Table 1:** Virulence of *Lecanicillium muscarium*, matrine, and combined *Lecanicillium muscarium* + matrine treatments against *Bemisia tabaci*.

Treatments		Mortality	LC_50_	95% C.I.	Slope	Chi-square	CTC*	Type of action
Matrine (mg/L)	0.005	19.07 ± 0.87 e	0.83 ± 0.09 a	0.54–1.13	0.09	16.65	—	—
	0.05	31.45 ± 1.35 d						
	0.5	48.29 ± 1.97 c						
	5	67.74 ± 2.68 b						
	15	84.36 ± 4.65 a						
	Control	4.49 ± 0.36 f						
*L. muscarium* (Conidia/mL)	1 × 10^4^	28.97 ± 1.04 e	0.11 ± 0.02 b	0.08–0.20	0.08	12.37	—	—
	1 × 10^5^	45.14 ± 2.65 d						
	1 × 10^6^	65.32 ± 3.47 c						
	1 × 10^7^	74.21 ± 3.78 b						
	1 × 10^8^	83.36 ± 5.09 a						
	Control	4.49 ± 0.37 f						
CT1	0.001 + 0.8 × 10^5^	61.23 ± 2.98 d	0.034 ± 0.01 d	0.02–0.04	0.47	18.97	125.99	Synergistic
	0.01 + 0.8 × 10^6^	74.63 ± 3.71 c						
	0.1 + 0.8 × 10^7^	82.54 ± 4.09 b						
	0.75 + 0.8 × 10^8^	98.31 ± 4.56 a						
	Control	4.45 ± 0.36 e						
CT2	0.0025 + 0.5 × 10^5^	54.13 ± 2.89 d	0.063 ± 0.01 c	0.05–0.08	0.34	15.74	200	Synergistic
	0.025 + 0.5 × 10^6^	61.89 ± 3.65 c						
	0.25 + 0.5 × 10^7^	79.76 ± 3.97 b						
	1.88 + 0.5 × 10^8^	95.06 ± 5.23 a						
	Control	4.45 ± 0.36 e						
CT3	0.004 + 0.2 × 10^5^	46.38 ± 3.21 d	0.21 ± 0.01 b	0.13–0.36	0.1	19.82	165.75	Synergistic
	0.04 + 0.2 × 10^6^	55.19 ± 2.85 c						
	0.4 + 0.2 × 10^7^	72.21 ± 3.65 b						
	4.5 + 0.2 × 10^8^	93.72 ± 4.56 a						
	Control	4.45 ± 0.36 e						

CT1, CT2 and CT3 are combined treatments of *Lecanicillium muscarium*, matrine mixed at different ratios; CTC stands for co-toxicity coefficients; Mortalities (% ± S.E.) mentioned for each treatment followed by different letters are significantly different (Tukey’s test, *P* < 0.01).

**Table 2:** Susceptibility of *Bemisia tabaci* to matrine after 24-h exposure to *Lecanicillium muscarium*.

Treatments		Mortality	LC_50_	95% C.I.	Slope	Chi-square	CTC	Type of action
Matrine (mg/L)	0.005	19.07 ± 0.87 e	0.97 ± 0.06 a	0.63–1.28	0.06	19.56	—	—
	0.05	27.41 ± 1.23 d						
	0.5	42.36 ± 1.89 c						
	5	63.49 ± 3.32 b						
	15	82.18 ± 4.16 a						
	Control	5.2 ± 0.37 f						
*L. muscarium* (Conidia/mL)	1 × 10^4^	28.97 ± 1.04 d	0.13 ± 0.001 b	0.10–0.20	0.07	11.23	—	—
	1 × 10^5^	42.8 ± 2.31 c						
	1 × 10^6^	61.03 ± 3.35 b						
	1 × 10^7^	72.36 ± 4.56 a						
	1 × 10^8^	79.81 ± 5.13 a						
	Control	5.3 ± 0.36 e						
CT4	0.005 + 1 × 10^5^	57.19 ± 3.81 d	0.02 ± 0.001 c	0.01–0.03	0.04	14.36	223	Synergistic
	0.05 + 1 × 10^6^	69.87 ± 3.56 c						
	0.5 + 1 × 10^7^	82.34 ± 4.23 b						
	5 + 1 × 10^8^	96.78 ± 6.31 a						
	Control	5.3 ± 0.36 e						

CT4 is combined treatment of *Lecanicillium muscarium* and, matrine mixed at 5:5 ratio; CTC stands for co-toxicity coefficients; Mortalities (% ± S.E.) mentioned for each treatment followed by different letters are significantly different (Tukey’s test, *P* < 0.01).

**Table 3:** Different concentrations of *Lecanicillium muscarium* and matrine used in the experiments.

Bioassay I	Bioassay II	Bioassay III						Bioassay IV	
*L. muscarium* (conidia/mL)	Matrine (mg/L)	CT1 (Mt* + Lm**2:8)		CT2 (Mt + Lm 5:5)		CT3 (Mt + Lm 8:2)		CT4 (Mt + Lm)	
1 × 10^4^	0.005 mg/L	0.001	0.8 × 10^5^	0.0025	0.5 × 10^5^	0.004	0.2 × 10^5^	0.005	1 × 10^5^
1 × 10^5^	0.05 mg/L								
1 × 10^6^	0.5 mg/L	0.01	0.8 × 10^6^	0.025	0.5 × 10^6^	0.04	0.2 × 10^6^	0.05	1 × 10^6^
1 × 10^7^	5 mg/L	0.1	0.8 × 10^7^	0.25	0.5 × 10^7^	0.4	0.2 × 10^7^	0.5	1 × 10^7^
1 × 10^8^	15 mg/L	0.75	0.8 × 10^8^	1.88	0.5 × 10^8^	4.5	0.2 × 10^8^	5	1 × 10^8^

*Mt = Matrine, **Lm = *Lecanicillium muscarium*.
